# The Impact of Fatigue on Sleep and Other Non-Motor Symptoms in Parkinson’s Disease

**DOI:** 10.3390/brainsci14040397

**Published:** 2024-04-19

**Authors:** Stefania Diaconu, Vlad Monescu, Rafaela Filip, Laura Marian, Cristian Kakucs, Iulia Murasan, K. Ray Chaudhuri, Dragos Catalin Jianu, Cristian Falup-Pecurariu, Bianca Opritoiu

**Affiliations:** 1 Department of Neurology, County Clinic Hospital, 500365 Brașov, Romania; stefi_diaconu@yahoo.com (S.D.); rafaela.filip@gmail.com (R.F.); valentina.irincu@yahoo.com (L.M.); iulia_murasan@yahoo.com (I.M.); crisfp100@yahoo.co.uk (C.F.-P.); 2 Faculty of Medicine, Transilvania University, 500036 Brașov, Romania; cristiankakucsnch@gmail.com (C.K.); opritoiubianca@yahoo.com (B.O.); 3 Faculty of Mathematics and Informatics, Transilvania University, 500036 Brașov, Romania; 4 Parkinson’s Foundation Centre of Excellence, King’s College Hospital, Denmark Hill, London SE5 9RS, UK; ray.chaudhuri@kcl.ac.uk; 5 Basic and Clinical Neuroscience, The Maurice Wohl Clinical Neuroscience Institute, Institute of Psychiatry, Psychology and Neuroscience, King’s College London, 5 Cutcombe Road, London SE5 9RX, UK; 6 Department of Neurosciences, “Victor Babes” University of Medicine and Pharmacy, 300041 Timisoara, Romania; dcjianu@yahoo.com; 7 “Pius Branzeu” Clinical Emergency County Hospital, 300736 Timisoara, Romania

**Keywords:** fatigue, Parkinson’s disease, sleep disturbances, anxiety, depression, quality of life

## Abstract

Fatigue is a common non-motor symptom in Parkinson’s disease (PD), but even so, it may still be underdiagnosed or misdiagnosed in current practice due to its non-specific manifestations. The aims of this study were to investigate the prevalence of fatigue in PD patients compared to healthy controls and to identify the main characteristics and associations of fatigue with other non-motor symptoms and the impact of fatigue on sleep disturbances in Parkinson’s disease. Materials and methods: case–control study in which 131 PD patients and 131 age- and sex-matched controls were enrolled. Main characteristics of fatigue, sleep, and other non-motor symptoms were assessed using specific validated questionnaires. Results: According to the Chalder fatigue scale, fatigue is more prevalent in PD patients (38.16%) compared to healthy controls (26.71%). Fatigue was identified in 46.54% of the PD patients using the Parkinson’s Fatigue Scale (PFS-16). PD patients with fatigue presented a worse motor status, more sleep disturbances (insomnia, daytime sleepiness), a broader spectrum of non-motor symptoms (pain, anxiety, urinary disturbances), worse cognitive performances, a lower level of happiness, and worse quality of life compared to PD patients without fatigue. Conclusion: Fatigue is a common symptom of PD and needs to be assessed, considering its consequences on quality of life. Sleep disturbances have a great influence over fatigue in PD patients.

## 1. Introduction

Fatigue is a complex symptom, characterized mainly by an overwhelming feeling of tiredness, exhaustion, or lack of energy [1]. Considering that certain non-motor symptoms are better expressed in some patients diagnosed with Parkinson’s disease (PD), the non-motor subtyping is reasonable from a clinical point of view [2]. The *Park fatigue* subtype has been previously described [2] and recognised through positron emission tomography (PET) studies that demonstrated limbic serotonergic deficit in this group of patients [3]. According to a meta-analysis, about half of PD patients may experience fatigue [4]. However, there may be a large overlap between the clinical manifestations of fatigue with those of depression and sleepiness; therefore, sometimes it may be difficult for the patient—but also for the clinician—to differentiate between these conditions, especially in advanced stages. Consequently, fatigue may be underdiagnosed, even if it is associated with a significant negative impact on patients’ quality of life [5].

According to a longitudinal study conducted over a period of 9 years, which initially involved 51 PD patients—at the end of the study, 21 patients remained—fatigue is a persistent symptom associated with depression, which tends to worsen over time. However, other co-factors (such as age or medication) can influence this tendency of evolution [6].

The neurodegenerative processes in PD, medication, motor symptoms, and circadian rhythm alteration may impair sleep by changing its normal architecture (reduced sleep time/efficiency, diminished percentages of N2 stage and rapid eye movement stage, increased apnoea/hypopnoea index, etc.) [7]. Therefore, the main sleep disturbances (such as insomnia, excessive daytime sleepiness, restless legs syndrome, rapid eye movement sleep behaviour disorder, sleep apnoea) and the sleep quality should be included in clinical routine examination [8].

Fatigue can have several correlations with sleep disturbances in PD as well. One polysomnographic study demonstrated lower percentages of rapid eye movement (REM) sleep in PD patients with severe fatigue compared to those with mild fatigue; in addition, REM sleep behaviour disorder (RBD) was a significant predictor of fatigue in this study [9]. According to a questionnaire-based study which was part of the International Nonmotor Scale validation project, fatigue correlated significantly with depression, anxiety, and sleep dysfunction [10]. Other identified associations of fatigue were excessive daytime sleepiness (EDS), anxiety, and apathy [11]. Disrupted sleep during the night-time may result in fatigue during the day; nocturia, a common non-motor symptom of PD, was shown to be associated with sleep fragmentation and fatigue [12]. Restless legs syndrome is another non-motor symptom which may have correlations with fatigue [13,14]. Poor sleep quality was a commonly reported trigger of fatigue in PD patients [15]. However, the complex interrelation between fatigue, sleep, and other non-motor symptoms has been scarcely explored in previous studies. The degeneration of the serotoninergic pathways and the dysfunction of the limbic–cortical circuitry may explain the correlations between fatigue, sleep disturbances, apathy, and anxiety [4]. Neuroinflammation may be a link between fatigue and RBD. In a study conducted on 102 PD patients, higher levels of α-synuclein oligomer were found in the cerebrospinal fluid of fatigued patients [16], while in a previous study, high levels of α-synuclein oligomer were found in patients with RBD [17]. Even if fatigue can be related to EDS, mental fatigue may be a persistent independent symptom that progresses along with the neurodegenerative processes [18]. However, the common brain areas involved in modulating both sleep/wake regulation and fatigue (basal forebrain, brainstem, thalamus, and hypothalamus) may support the association between EDS and fatigue [3,11]. Medication may also have an important influence on fatigue and sleep disturbances. Levodopa may have a beneficial effect on fatigue in PD patients [19]. Higher levodopa equivalent daily dose (LEDD) was noticed in PD patients with fatigue in some studies [9,20], while similarly, LEDD is known to be associated with EDS [21] and RBD [22]. According to one study, patients who received a combination of levodopa/dopamine agonists presented more EDS compared to those under levodopa monotherapy, but no differences were noted regarding fatigue between these two groups [23]. Benzodiazepines may help in alleviating certain sleep disturbances (RBD, insomnia) [24] but may induce fatigue and somnolence as primary side effects [25], or excitement, restlessness, or aggressivity as paradoxical reactions [26].

Several interventions were proposed for the management of fatigue in PD, including medication (methylphenidate, modafinil, and rasagiline) and physical activity [27]. Opicapone may also improve sleep and fatigue in PD patients [28].

The aims of this study were as follows: (a.) to explore the prevalence of fatigue among PD patients and healthy controls; (b.) to describe the characteristics and main associated factors of fatigue in PD patients and their influence on sleep; (c.) to explore the influence of sleep disturbances on fatigue in PD patients.

## 2. Materials and Methods

### 2.1. Patients and Study Design

In this case–control study conducted within the Department of Neurology of the County Clinic Hospital, Transilvania University of Braşov, 131 PD patients and 131 age- and sex-matched controls were enrolled. The inclusion criteria for the PD group were as follows: (a.) the diagnosis of PD, based on the Movement Disorders Society (MDS) criteria, regardless of the severity or duration of the illness; (b.) age ≥ 18 years; (c.) voluntary participation agreement by signing the informed consent. The same inclusion criteria, except for the PD diagnosis, were applied for the control group. Exclusion criteria for both groups were as follows: (a.) atypical or secondary parkinsonism; (b.) diagnosis of severe psychiatric or neurological conditions; (c.) severe speech disorders or cognitive impairments that might interfere with the quality of clinical examination and the ability to complete the administered questionnaires. According to the main objectives of this study, the prevalence of fatigue was compared between PD patients and healthy individuals, and then a detailed comparison was conducted between PD patients with fatigue (PD + fatigue) vs. PD patients without fatigue (PD-fatigue) in relation to sleep and other motor and non-motor features.

### 2.2. Clinical Assessment

All participants filled in a standardized case report form that included questions related to demographic data (e.g., age, gender, educational level), pre-existing medication, and sleep habits (e.g., usual bedtime, estimated number of hours of sleep/night). For PD patients, additional collected data included age at PD onset, duration of the illness, Hoehn and Yahr stage (in both ON and OFF states), and the levodopa equivalent daily dose (LEDD). Subsequently, validated scales and questionnaires for assessing the motor status and other various symptoms such as sleep, fatigue, and other non-motor symptoms were completed by either the patient or the examiner.

This study was approved by the Ethics Committee of University Transilvania of Brasov (1.11/01/2019), and it was conducted in accordance with the principles of the Declaration of Helsinki.

### 2.3. Questionnaires and Rating Scales

The motor status of the PD patients was assessed using the MDS-UPDRS (MDS Unified Parkinson’s Disease Rating Scale) scale, part III [29], SCOPA (Scales for Outcomes in Parkinson’s disease)—specifically the motor component scale [30], the Clinical Impression of Severity Index (CISI) [31], and by the Hoehn and Yahr (H&Y) stage.

In order to evaluate fatigue, the following scales were applied: the Fatigue Impact Scale (FIS) and the Chalder assessment tool for the control group, and FIS, Chalder, and Parkinson Fatigue Scale (PFS-16) for patients with PD. The Fatigue Impact Scale (FIS) [32] is an assessment tool composed of 40 items that measure the impact of fatigue in three domains—physical functionality (10 items), cognitive functionality (10 items), and psychosocial functionality (20 items)—over the last month [33]. For each item, response options can vary on a Likert scale from “0: no problems” to “4: extreme problems”. Higher scores suggest greater severity of fatigue [33]. The Chalder Fatigue Questionnaire was designed to characterize fatigue in general population [34,35]. It consists of 11 items for which a total score is obtained and is composed of two domains—physical fatigue and mental fatigue. The interpretation of the obtained score can be conducted in two ways: by summing individual responses scored from 0 to 3, resulting in a total score that can range from 0 to 33, or by calculating responses in a binary manner, with a total score ranging from 0 to 11. When using the Likert scale scoring, a total score between 29 and 33 may indicate clinically significant fatigue. For the binary scoring method, a value ≥ 4 is suggestive of fatigue [36]. The Parkinson Fatigue Scale (PFS-16) [37] was specifically designed for PD patients and consists of 16 self-assessment items related to fatigue and its consequences. Responses for each item can vary on a scale from “1: strongly disagree” to “5: strongly agree”. Fatigue is identified if the overall average score on the PFS-16 scale is ≥3.3, as indicated by previous studies [37]. PFS-16 evaluates the physical dimension of fatigue and its impact on daily function, being a scale designed for screening purposes [38]. PFS-16 was applied only for the PD patients, while Chalder and FIS were used to assess fatigue in both groups (PD and control group).

Several questionnaires were used in this study for sleep assessment: Parkinson’s Disease Sleep scale-2 (PDSS-2) [39], Scales for Outcomes in Parkinson’s Disease (SCOPA)–Sleep [40], Pittsburgh Sleep Quality Index (PSQI) [41], Insomnia Severity Index (ISI) [42], Athens Insomnia Scale (AIS) [43], Epworth Sleepiness Scale (ESS) [44], and International Restless Legs Scale (IRLS) [45]. PDSS-2 and SCOPA–sleep were only used for sleep assessment in the PD group, while PSQI, ISI, AIS, ESS, and IRLS were applied in both groups.

Quality of life of the PD patients was explored using The Parkinson’s Disease Questionnaire (PDQ-39) [46].

The examination of cognitive function was conducted in both groups based on the Mini-Mental State Examination (MMSE) and the Montreal Cognitive Assessment (MoCA) scales. The latter is recommended as a more specific evaluation for assessing cognitive capacity in patients with neurodegenerative diseases [47,48].

The non-motor symptoms of PD patients were evaluated according to the Non-Motor Symptoms Questionnaire–NMSQ [49], Non-Motor Symptoms Scale (NMSS [50]) and The International Parkinson and Movement Disorder Society–Non-Motor Rating Scale (MDS-NMS) [51]. Pain was assessed in the PD group using King’s Parkinson’s Disease Pain Questionnaire (KPPQ) [52] and King’s Parkinson’s disease Pain Scale (KPPS) [53]. Urinary function was investigated in PD patients using the Urogenital Distress Inventory (UDI-6) [54] and the Overactive Bladder Questionnaire (OABq)–short form [55] scales.

The following scales were used in the PD group to determine the levels of anxiety and depression and the psychological characteristics of the patients: Hospital Anxiety and Depression Scale [56], Parkinson’s Anxiety Scale (PAS) [57], Self-Compassion Scale (SCS) [58], Penn State Worry Questionnaire (PSWQ) [59], and Oxford Happiness Questionnaire [60].

### 2.4. Data Analysis

The recorded data were analysed using RStudio and IBM SPSS for Windows, version 26.0. Descriptive data were presented as mean ± standard deviation (SD). A probability value (*p*) < 0.05 was considered statistically significant. The distribution of the sample was determined using the Shapiro–Wilk test. The Shapiro–Wilk test is a statistical test used to assess the normality of a dataset, determining whether it follows a normal distribution.

The Shapiro–Wilk test is a justified and widely used method for assessing the normality of data distributions due to its sensitivity, applicability to small sample sizes, robustness, and support from statistical literature and guidelines. It provides a reliable tool for evaluating whether the data conform to the assumptions of normality required for many statistical analyses [61].

Pearson correlation was used to determine the correlations between various parameters. Chi-square, Fisher, and Mann–Whitney U tests were employed to compare characteristics between groups. Logistic regression models were applied to identify predictors for various analysed parameters, being considered statistically significant those models that presented statistical significance (*p* < 0.05). The Kruskal–Wallis test was used to compare the means of subscales in the examined groups.

We tested the statistical correlation between the variables and calculated the corresponding *p*-values which indicate the statistical significance of the observed correlation. For the studied groups, the median, average, and standard deviation were calculated. Also, the *p*-value in a *t*-test is calculated to determine the statistical significance of the difference between the means of the two corresponding studied groups.

## 3. Results

### 3.1. Prevalence of Fatigue in PD Patients Compared to Controls

Fatigue (according to a Chalder scale score ≥ 4) is more frequent in PD patients (38.16%) compared to controls (26.71%). When assessing the mean scores of the individual items of the Chalder scale, patients with PD have also markedly higher scores for all items compared to the control group, with items 1–9 showing statistical significance.

### 3.2. Main Characteristics of Fatigue in PD Patients

1. Fatigue and motor symptoms

Table 1 represents the distribution of mean scores and standard deviation (SD) for the scales that evaluate fatigue in relation to PD severity (according to H&Y staging). Progressively higher scores for the FIS, PFS-16, and Chalder scales can be observed in patients with advanced stages compared to those in early stages (*p* < 0.001 for FIS and PFS-16, *p* = 0.10 for Chalder).

PD patients were evaluated using the PFS-16 scale, and those with a total score ≥ 3.3 were considered PD patients + fatigue (61 patients, 46.54%), while those with scores < 3.3 were classified as PD patients–fatigue (70 patients, 53.43%). The clinical characteristics of these two patient categories are presented in Table 2. No statistically significant differences were observed regarding age at the onset of PD, age at the time of assessment, duration of PD, gender, or LEDD between the two groups. More PD patients + fatigue (23, 37.7%) were undergoing benzodiazepine treatment compared to those without fatigue (14, 20%, *p* = 0.04); otherwise, no significant differences regarding medication were noticed. Patients with fatigue have fewer estimated hours of sleep/night than those without fatigue (6.04 ± 1.76 h vs. 6.90 ± 1.41, *p* = 0.002). The MDS-UPDRS, SCOPA–motor part III and CISI total scores are higher in patients with PD + fatigue compared to patients with PD–fatigue (*p* < 0.001).

2. Fatigue and sleep in PD patients

**Figure 1:**
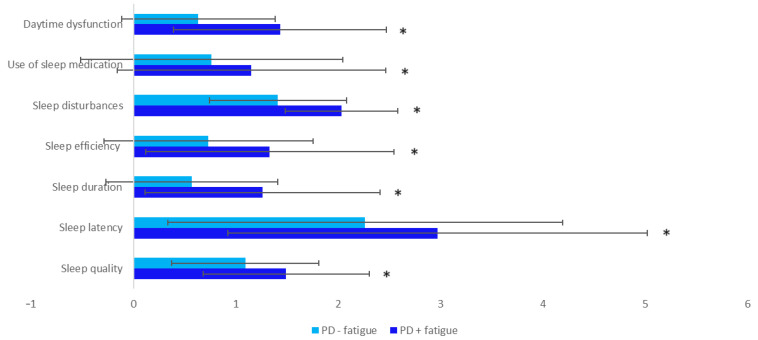
Average scores of the individual components of the PSQI scale, in patients with and without fatigue. Statistically significant results are marked with an asterisk (*). PSQI: Pittsburgh Sleep Quality Index.

Figure 1 depicts the average PSQI sub-scores in patients with and without fatigue. Patients with fatigue have higher values for all PSQI components compared to subjects without fatigue, with the following domains showing statistical significance: daytime dysfunction, sleep disturbances, sleep efficiency, sleep duration, sleep latency, and sleep quality.

Moreover, PD patients considered to be “bad sleepers” (PDSS-2 score ≥ 18) presented a higher level of fatigue than good sleepers (PDSS score < 18), according to the higher medium scores of the assessment scales for fatigue in ”bad sleepers” (FIS: 73.15 ± 22.21 vs. 53.59 ± 16.20, *p* < 0.001, PFS-16: 3.49 ± 0.96 vs. 2.13 ± 0.84, *p* < 0.001, Chalder: 14.80 ± 4.82 vs. 11.72 ± 1.75, *p* < 0.001).

Table 3 shows the average values of the various scales used to assess sleep in patients with PD and fatigue, compared to patients with PD without fatigue. Statistically significant data were recorded for all analysed scales. Patients with PD + fatigue have higher scores than those without fatigue in the following scales: SCOPA sleep (nocturnal and daytime symptom components), ESS, ISI, AIS, and IRLS.

3. Fatigue and quality of life in PD patients

**Figure 2:**
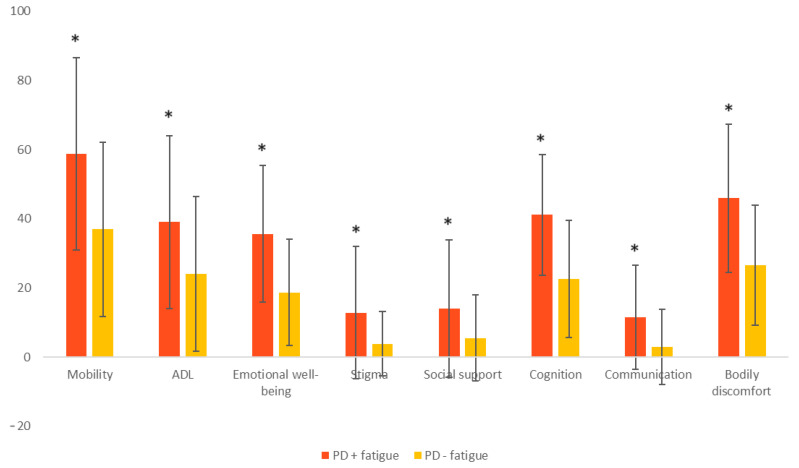
Distribution of the mean scores of the components of the PDQ-39 scale in patients with PD and fatigue, compared to patients with PD without fatigue. Statistically significant results are marked with an asterisk (*). ADL: activities of daily living; PD: Parkinson’s disease.

The analysis of the quality of life according to the PDQ-39 score in patients with PD with and without fatigue is illustrated in Figure 2. Patients with PD and fatigue present statistically significant higher scores than those without fatigue for all domains of the PDQ-39 scale.

4. Fatigue and other non-motor symptoms

The spectrum of the non-motor symptoms in the PD group in relation with fatigue was analysed using the following scales: NMSQ, NMSS, and MDS-NMS. Other non-motor symptoms that were assessed with specific validated scales in the PD group were anxiety, depression, cognition, and urinary function. We also evaluated self-compassion, happiness, and worry in PD patients with fatigue and without fatigue. These results (differentiated for the PD + fatigue and PD-fatigue) are shown in Table 4. PD patients + fatigue presented significantly higher mean scores for the following scales compared to PD patients-fatigue: NMSQ, NMSS, KPPQ, KPPS, HADS (including HADS-A and HADS-D subscales), PAS, OHQ, and PSWQ. On the contrary, patients with PD + fatigue present lower scores for the MMSE, MoCA, SCS, and OHQ scales compared to patients with PD without fatigue.

**Figure 3:**
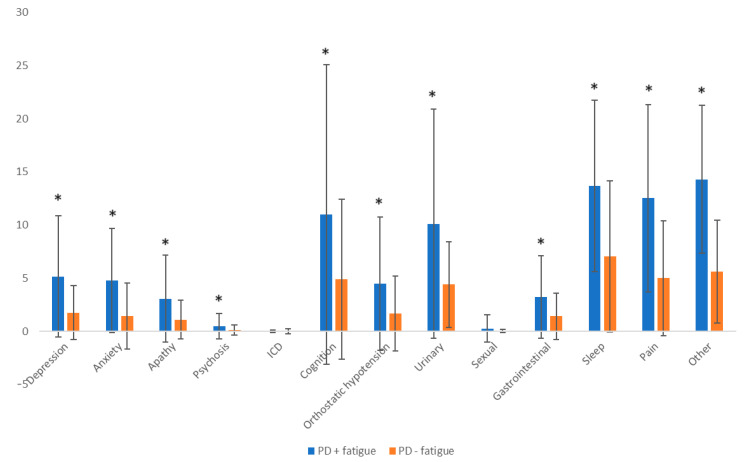
Mean total scores of MDS-NMS scale domains in patients with PD + fatigue compared to patients with PD–fatigue. Statistically significant results are marked with an asterisk (*). ICD: impulse-control disorder; PD: Parkinson’s disease.

The total average scores of the sub-domains of the MDS-NMS scale in relation to the presence or absence of fatigue are represented in Figure 3. For the following domains, statistically significantly higher values were revealed in patients with PD and fatigue compared to those without fatigue: depression, anxiety, apathy, psychosis, cognition, orthostatic hypotension, urinary, gastrointestinal, sleep, pain, and other symptoms.

5. Factors associated with fatigue in PD patients

The Pearson test was used to analyse correlations between PFS-16 and clinical characteristics. As can be seen in Table 5, weak positive correlations were found for ESS. Moderate positive correlations were recorded between PFS-16 and MDS-UPDRS motor part, PSQI, PDSS-2, and PDQ-39.

Binary logistic regression analysis was performed to identify factors associated with fatigue in patients with PD (based on a PFS-16 score ≥ 3.3). As is shown in Table 6, the components that reached statistical significance (*p* < 0.05) were the duration of the disease, the motor status (according to the MDS-UPDRS part III assessment), and the presence of sleep disorders (according to the assessment of PSQI, PDSS-2, SCOPA-sleep components of nocturnal and daytime symptoms, insomnia and EDS are the factors associated with fatigue in an independent assessment model).

A proposed model in which age, motor status, the presence of sleep disorders (according to PDSS-2) and insomnia (according to ISI) are analysed cumulatively suggests that fatigue is associated with motor status (according to MDS-UPDRS part III) and insomnia, according to AIS (Table 7).

## 4. Discussion

Fatigue is a common symptom in Parkinson’s disease, with an estimated prevalence ranging from 33% to 70% [62]. However, considering the various individual perceptions regarding fatigue and numerous confounding symptoms (somnolence, apathy, depression, etc.), it may remain underdiagnosed [63]. Based on the mean total score of the PFS-16 scale, fatigue was identified in 61 (46.54%) of the PD patients enrolled in the present study. Some researchers suggest that fatigue could be a special subtype of PD, considering the correlations between fatigue and altered sleep architecture [9]. Several neuromediators, including dopamine, contribute to the physiological transition between wakefulness and sleep stages [64]. In PD, the neurodegenerative processes that impair the neurotransmitter function and the side effects of dopaminergic drugs are the two main causes for the occurrence of sleep disturbances [65]. PD patients with fatigue are more prone to apathy [66], with other important associations being with depression [67], cognitive dysfunction [68], and reduced quality of life [63].

In this study, fatigue had a higher prevalence in PD patients compared to the control group (38.16% vs. 26.71%). Similarly, in the study conducted by Rabo et al., PD patients showed more fatigue compared to participants in the control group [69]. Approximately one-third of PD patients consider fatigue as one of the three most bothersome symptoms [70].

In the present study, for each item of the Chalder scale, higher mean values of the sub-scores were found in PD patients (indicating more fatigue) compared to controls. The phenomenology and aetiology of fatigue has been incompletely evaluated until now.

Fatigue is also more expressed in patients with advanced PD stages compared to those with early stages, as shown by higher PFS-16, FIS, and Chalder scores in patients with higher H&Y stages. More patients with PD + fatigue were undergoing benzodiazepine medication compared to patients with PD–fatigue. Also, motor status is more advanced in patients with PD + fatigue compared to patients without fatigue, according to MDS-UPDRS part III, SCOPA–motor part, and CISI scores (*p* < 0.001).

A study on a representative group of PD patients (2100) carried out in China (Parkinson’s Disease & Movement Disorders Multicentre Database and Collaborative Network in China (PD-MDCNC)) identified fatigue in more than one third of the investigated patients (36.8%, according to the PFS-16 scale). Compared to patients in whom fatigue was not identified, the ones with fatigue were older, had a longer disease duration, higher LEDD, and more pronounced motor and non-motor symptoms. UPDRS–part 3 and NMSS total scores, the presence of EDS, and motor fluctuations (wearing-off) were significantly associated with fatigue in the previously mentioned study [71]. Similarly, another study, also conducted in China on a group of 222 patients with PD, reported a frequency of fatigue of 59.46%. Patients with fatigue had a longer disease duration and more severe motor symptoms (except tremor), more depressive symptoms, and more sleep disturbances than patients without fatigue. Severity of sleep dysfunctions was an independent factor for fatigue [72].

Another study that included 114 patients with idiopathic PD identified fatigue in more than half of the evaluated patients (57.9%) based on Fatigue Severity Scale total scores. According to the results of this research, greater intensity of fatigue was correlated with LEDD and depression [20].

There were no significant gender differences in fatigue (according to FIS, PFS-16, and Chalder evaluations) in the present study. In contrast to this result, females reported fatigue more frequently, according to one study [73]. Likewise, Hu et al. reported that females showed more fatigue than males [74].

According to the results of the present study, the PFS-16 score shows moderate positive correlations with the MDS-UPDRS scores (the motor part), PSQI, PDSS-2, and PDQ-39 and weak positive correlations with ESS. Patients with PD + fatigue reported fewer total estimated hours of sleep per night compared to those without fatigue. Pain (assessed with the KPPQ and KPPS scales), anxiety, and depression (assessed with the HADS and PAS scales) are more pronounced in patients with fatigue. Moreover, PD patients with fatigue presented higher worry and less happiness than PD patients without fatigue. PD patients with fatigue also present more pronounced restless legs syndrome (RLS) symptoms than those without fatigue (IRLSSG score 5.61 ± 7.98 vs. 2.49 ± 6.26, *p* = 0.013).

The association between fatigue and urinary disorders has been insufficiently described in previous studies. In a study conducted on 81 PD patients in which the PFS (PD Fatigue Scale) and FSS (Fatigue Severity Scale) scales were used for the assessment of fatigue, significant correlations were observed between fatigue, non-motor symptoms (assessed by the NMSS scale), and motor severity (assessed by the H&Y score and the UPDRS scale–part III). The NMSS domains that can predict fatigue were found to be domain 3 (depression/anxiety) and domain 2 (sleep disturbances). In addition, there was an observed escalation in fatigue severity as the range of non-motor symptoms expanded [73].

Using a binary logistic regression model which analysed factors independently, disease duration, motor status, sleep disorders, insomnia, non-motor symptoms, and EDS were identified in the present study as fatigue-associated factors. In another model in which the factors were analysed cumulatively, only an association between fatigue, motor status, and insomnia was noted. Another study conducted in Norway (the ParkWest project), which included 184 de novo patients with PD, assessed fatigue using the Fatigue Severity Scale. According to the results of this study, fatigue correlated significantly with motor severity (assessed by UPDRS part III), sleep disturbances, depressive symptoms, apathy, and reduced processing speed and visuospatial skills [68].

The results of the present study suggest that fatigue may have an impact on the quality of life in PD patients, considering the higher total values of the PDQ-39 scale and all its sub-domains in patients with PD + fatigue compared to those without this symptom. In line with these results, the negative impact of fatigue on quality of life has also been demonstrated by other studies. Patients with fatigue reported more discomfort in relation to emotional well-being [75], mobility, and PDQ-39 total score [75,76].

A study including 361 PD patients and 100 controls analysed the correlations between fatigue, sleep disturbances, and quality of life using, among other scales, PDSS-1, PDQ-39, and PFS-16. A PFS-16 score ≥ 3.3 (suggestive of fatigue) was identified in 41.8% of patients with PD, compared to 5% of those in the control group. Sleep disturbances and a reduced quality of life were the independent factors associated with fatigue. Moreover, fatigue was significantly correlated with those items of PDSS-1 related to restless sleep and urinary incontinence [76]. There were no correlations between fatigue and depression in this study [76].

The consequences of fatigue are, among others, sleep disorders and various non-motor disorders. The PD patients with fatigue which were evaluated in the present study presented more EDS (according to ESS scores) and insomnia (according to ISI and AIS scores) than PD patients without fatigue. A study in which 128 PD patients were enrolled and evaluated by various scales (Beck Depression Inventory, ISI, FSS, ESS) demonstrated that fatigue correlates with insomnia and EDS [77]. Fatigue may have an influence on insomnia due to the fact that it may contribute to decreased physical activity during the day [77]. The severity of fatigue may also correlate with sleep dysfunction. In a study conducted on 232 PD patients, which were divided into two groups according to the severity of fatigue, the Fatigue Severity Scale was used for assessment. Patients with more severe fatigue had a longer duration of PD, more advanced stages (according to the H&Y scale), higher LEDD, and also more pronounced depressive symptoms, anxiety, and EDS compared to patients with PD without fatigue [9].

In the current study, the PD patients considered to be “bad sleepers” (PDSS-2 score ≥ 18) presented more fatigue than good sleepers (PDSS score < 18), an aspect suggested by the higher total scores of the scales that evaluate fatigue (FIS, PFS-16, Chalder) in “bad sleepers” (*p* < 0.001). A longitudinal study of the PALS (The Early Parkinson’s Disease Longitudinal Singapore) cohort that included 109 patients with PD compared the clinical characteristics of the participants considered “good sleepers” (based on a PSQI score ≤ 5) and “bad sleepers” (PSQI > 5) at the initial assessment and 1 year later. Initially, “bad sleepers” had more anxiety (according to the HADS assessment) than “good sleepers”. At the follow-up evaluation, “bad sleepers” had more fatigue (based on the Fatigue Severity Scale scores) and depression (based on the GDS-Geriatric Depression Scale score) compared to “good sleepers”, indicating that the reduced quality of sleep was initially associated with more anxiety, and as the disease progressed, with fatigue and depression [78].

Cognitive capacity may also be influenced by fatigue. In this study, patients with fatigue present lower scores (meaning greater cognitive dysfunction) of the MMSE and MoCA tests compared to patients without this symptom. These results were also confirmed by the MDS-NMS scale, as statistically significantly higher scores were observed in patients with PD and fatigue for all non-motor domains (except for the impulse control disorder and sexual domains) compared to patients with PD without fatigue. Cognition may be associated with fatigability, particularly visuospatial dysfunction [79]. Dysfunctions of the basal ganglia and medial frontal cortex can generate fatigue by disrupting the circuits that modulate volitional effort [79]. Patients with moderate/severe fatigue reported more sleep disturbances and also more apathy than controls [68]. On the contrary, a study that included 81 PD patients without antiparkinsonian treatment did not observe correlations between fatigue and cognitive disorders. Among the investigated patients, 15% presented bothersome fatigue (based on the PFS scale), which correlated with episodic anxiety, cognitive apathy, and sleepiness, but not with cognitive dysfunction [11].

## 5. Limitations

We acknowledge that our study has several limitations. Firstly, this was a questionnaire-based study; therefore, objective tools such as polysomnography were lacking. Secondly, the urinary function of the patients was not assessed by a specialist in the field, using objective means. In this study, we did not include other paraclinical data, which would be useful for excluding other possible causes of fatigue.

## 6. Conclusions

Fatigue is a common symptom in PD patients. It progresses as the severity of the disease increases and it has complex interactions with sleep and other non-motor symptoms (such as pain, anxiety, and depression). Considering all these facts and the negative consequences of fatigue on patients’ quality of life, early identification of fatigue is mandatory, and multidisciplinary and personalized approaches should be provided in order to cover its multifaceted aspects.

## Figures and Tables

**Table 1:** Average scores of fatigue assessment scales (FIS, PFS-16, Chalder) according to H&Y stage of patients with PD. Bold values denote statistical significance (*p* < 0.05).

	H&Y Stage I	H&Y Stage II	H&Y Stage III	H&Y Stage IV and V	*p*
**FIS total score, mean (SD)**	46.29 (12.50)	62.79 (21.39)	73.26 (19.38)	92.25 (17.72)	**<0.001**
**PFS-16 total score, mean (SD)**	2.23 (1.15)	2.90 (1.12)	3.37 (1.00)	4.03 (0.50)	**<0.001**
**Chalder total score, mean (SD)**	11.29 (1.25)	13.47 (4.01)	14.12 (5.07)	17.42 (4.08)	**0.010**

FIS: Fatigue Impact Scale; PFS16: Parkinson’s Disease Fatigue Scale; SD: standard deviation.

**Table 2:** Clinical characteristics of patients with PD and fatigue, compared to patients with PD without fatigue. Bold values denote statistical significance (*p* < 0.05).

	PD + Fatigue (*n* = 61, 46.54%)	PD–Fatigue (*n* = 70, 53.43%)	*p* Value
Age at PD onset—years, mean (SD)	69.48 (10.28)	69.14 (10.00)	0.852
Age at assessment—years, mean (SD)	75.43 (8.86)	73.54 (9.06)	0.233
Sex, female, *n* (%)	34 (55.7)	33 (47.1)	0.42
PD duration—years, mean (SD)	5.80 (4.43)	4.42 (3.94)	0.064
Melatonin treatment, *n* (%)	3 (4.9)	4 (5.7)	1.00
**Benzodiazepine treatment, *n* (%)**	23 (37.7)	14 (20.0)	**0.04**
Non-benzodiazepine treatment, *n* (%)	4 (6.6)	4 (5.7)	1.00
Other antidepressive medication, *n* (%)	7 (11.5)	2 (2.9)	0.11
LEDD—mg, mean (SD)	567.49 (405.55)	436.06 (358.38)	0.051
Estimated sleep latency, minutes, mean (SD)	43.02 (37.46)	35.43 (35.63)	0.243
**Estimated hours of sleep/night, mean (SD)**	6.04 (1.76)	6.90 (1.41)	**0.002**
**MDS-UPDRS total score, mean (SD)**	38.41 (13.92)	27.51 (11.16)	**<0.001**
**SCOPA–motor total score, mean (SD)**	26.30 (9.16)	18.59 (7.80)	**<0.001**
**CISI total score, mean (SD)**	9.57 (3.71)	6.71 (2.91)	**<0.001**

CISI: Clinical Impression of Severity Index; LEDD: levodopa equivalent daily dose; MDS-UPDRS: Movement Disorder Society-Unified Parkinson’s Disease Rating Scale; PD: Parkinson’s disease; SCOPA: Scales for Outcomes in Parkinson’s Disease; SD: standard deviation.

**Table 3:** Distribution of the average scores of the various sleep scales analysed in patients with PD and fatigue compared to patients with PD without fatigue. Bold values denote statistical significance (*p* < 0.05).

	PD + Fatigue (*n* = 61, 46.54%)	PD–Fatigue (*n* = 70, 53.43%)	*p*
**SCOPA–sleep NS total score, mean (SD)**	8.08 (3.32)	5.29 (3.22)	**<0.001**
**SCOPA–sleep DS total score, mean (SD)**	6.03 (3.85)	3.33 (2.87)	**<0.001**
**ESS total score, mean (SD)**	11.00 (5.28)	7.33 (5.16)	**<0.001**
**ISI total score, mean (SD)**	13.80 (5.37)	7.93 (5.09)	**<0.001**
**AIS total score, mean (SD)**	11.11 (4.43)	5.94 (4.26)	**<0.001**
**IRLS total score, mean (SD)**	5.61 (7.98)	2.49 (6.26)	**0.013**

AIS: Athens Insomnia Scale; DS: daytime symptoms; ESS: Epworth Sleepiness Scale; IRLS: International Restless Legs Scale; ISI: Insomnia Severity Index; SCOPA: Scales for Outcomes in Parkinson’s Disease; SD: standard deviation.

**Table 4:** Distribution of the average scores of the scales used to analyse other non-motor symptoms in patients with PD and fatigue, compared to patients with PD without fatigue. Bold values denote statistical significance (*p* < 0.05).

	PD + Fatigue (*n* = 61, 46.54%)	PD-Fatigue (*n* = 70, 53.43%)	*p*
General spectrum of the non-motor symptoms			
NMSQ total score, mean (SD)	11.72 (4.58)	6.69 (4.17)	**<0.001**
NMSS total score, mean (SD)	64.64 (34.32)	28.63 (19.37)	**<0.001**
Pain			
KPPQ total score, mean (SD)	6.20 (3.33)	3.21 (2.58)	**<0.001**
KPPS total score, mean (SD)	24.43 (14.77)	9.26 (8.68)	**<0.001**
Cognition			
MMSE, mean (SD)	26.46 (3.40)	28.01 (3.21)	**0.008**
MoCA, mean (SD)	22.13 (5.78)	24.57 (5.05)	**0.011**
Urinary function			
OABQ total score, mean (SD)	44.43 (19.89)	32.94 (14.50)	**<0.001**
UDI-6 total score, mean (SD)	41.94 (19.66)	31.90 (13.19)	**0.001**
Anxiety, depression			
HADS total score, mean (SD)	18.31 (7.57)	10.76 (5.99)	**<0.001**
HADS-A total score, mean (SD)	7.26 (3.88)	3.67 (2.90)	**<0.001**
HADS-D total score, mean (SD)	11.05 (4.17)	7.04 (3.61)	**<0.001**
PAS total score, mean (SD)	10.03 (7.14)	3.89 (5.88)	**<0.001**
Happiness, self-compassion, worry			
OHQ total score, mean (SD)	3.47 (0.85)	4.20 (0.76)	**<0.001**
SCS total score, mean (SD)	3.05 (0.72)	3.58 (0.66)	**<0.001**
PSWQ total score, mean (SD)	46.61 (8.28)	39.93 (5.99)	**<0.001**

HADS: Hospital Anxiety and Depression Scale; HADS-A: Hospital Anxiety and Depression Scale–Anxiety; HADS-D: Hospital Anxiety and Depression Scale–Depression; KPPQ: King’s Parkinson’s disease Pain Questionnaire; KPPS: King’s Parkinson’s disease Pain Scale; MMSE: The Mini Mental State Examination; MoCA: Montreal Cognitive Assessment; NMSQ: Non-Motor Symptoms Questionnaire; NMSS: Non-Motor Symptoms Scale; OABq: Overactive Bladder Questionnaire; OHQ: Oxford Happiness Questionnaire; PAS: PAS: Parkinson Anxiety Scale; PSWQ: Penn State Worry Questionnaire; SCS: Self-Compassion Scale; UDI-6: Urinary Distress Inventory; SD: standard deviation.

**Table 5:** Pearson analysis of correlations between the PFS-16 score and various other characteristics/scores. Bold values denote statistical significance (*p* < 0.05).

	*r*	*p*
**MDS–UPDRS part III**	0.429	<0.001
**PSQI**	0.495	<0.001
**PDSS-2**	0.545	<0.001
**PDQ39**	0.549	<0.001
**ESS**	0.366	<0.001
**HADS**	0.526	<0.001
**HADS–A**	0.465	<0.001
**HADS–D**	0.526	<0.001

ESS: Epworth Sleepiness Scale; Hospital Anxiety and Depression Scale–Anxiety; HADS-D: Hospital Anxiety and Depression Scale–Depression; MDS-UPDRS: Movement Disorder Society-Unified Parkinson’s Disease Rating Scale; PDQ-39: Parkinson’s Disease Questionnaire-39; PDSS-2: Parkinson’s Disease Sleep Scale–2; PSQI: Pittsburgh Sleep Quality Index.

**Table 6:** Logistic regression analysis of parameters to identify the factors associated with fatigue. Bold values denote statistical significance (*p* < 0.05).

Clinical Characteristics	Odds Ratio (95% CI)	*p*
Age	1.024 (0.985–1.065)	0.232
Sex	0.669 (0.335–1.334)	0.253
**Disease duration**	1.085 (1.023–1.151)	**0.007**
LEDD	1.001 (1.000–1.002)	0.055
**MDS-UPDRS part III**	1.072 (1.038–1.107)	**<0.001**
**PSQI**	1.219 (1.118–1.329)	**<0.001**
**PDSS-2**	1.125 (1.074–1.179)	**<0.001**
**SCOPA-sleep diurnal symptoms component**	1.284 (1.135–1.453	**<0.001**
**SCOPA-sleep nocturnal symptoms component**	1.295 (1.148–1.461)	**<0.001**
**ESS**	1.142 (1.063–1.227)	**<0.001**
**AIS**	1.287 (1.172–1.143)	**<0.001**

AIS: Athens Insomnia Scale; ESS: Epworth Sleepiness Scale; LEDD: levodopa equivalent daily dose; MDS-UPDRS: Movement Disorder Society-Unified Parkinson’s Disease Rating Scale; PDSS-2: Parkinson’s Disease Sleep Scale–2; PSQI: Pittsburgh Sleep Quality Index; SCOPA: Scales for Outcomes in Parkinson’s Disease.

**Table 7:** Logistic regression analysis model of factors associated with fatigue. Bold values denote statistical significance (*p* < 0.05).

	*p*	Odds Ratio	95% CI
Age	0.279	0.973	(0.925–1.023)
MDS-UPDRS III	**0.006**	1.053	(1.015–1.092)
PSQI	0.374	1.062	(0.931–1.211)
AIS	**0.009**	1.201	(1.046–1.379)

AIS: Athens Insomnia Scale; MDS-UPDRS: Movement Disorder Society-Unified Parkinson’s Disease Rating Scale; PSQI: Pittsburgh Sleep Quality Index.

## Data Availability

The data presented in this study are available on reasonable request from the corresponding author. The data are not publicly available due to ethical concerns.
